# Betulin and Its Derivatives Reduce Inflammation and COX-2 Activity in Macrophages

**DOI:** 10.1007/s10753-022-01756-4

**Published:** 2022-10-25

**Authors:** Wojciech Szlasa, Sylwester Ślusarczyk, Izabela Nawrot-Hadzik, Renata Abel, Aleksandra Zalesińska, Anna Szewczyk, Natalia Sauer, Robert Preissner, Jolanta Saczko, Marcin Drąg, Marcin Poręba, Małgorzata Daczewska, Julita Kulbacka, Małgorzata Drąg-Zalesińska

**Affiliations:** 1 Faculty of Medicine, Wroclaw Medical University, Wroclaw, Poland; 2 Department of Pharmaceutical Biology and Biotechnology, Faculty of Pharmacy, Wroclaw Medical University, Wroclaw, Poland; 3 Institute of Physiology, Charité – Universitätsmedizin Berlin, corporate member of Freie Universität Berlin, Humboldt-Universität Zu Berlin, and Berlin Institute of Health, Berlin, Germany, Philippstrasse 12, 10115 Berlin, Germany; 4 Department of Molecular and Cellular Biology, Faculty of Pharmacy, Wroclaw Medical University, Wroclaw, Poland; 5 Faculty of Pharmacy, Wrocław Medical University, Wroclaw, Poland; 6 Science-IT and Institute of Physiology, Charité – Universitätsmedizin Berlin, corporate member of Freie Universität Berlin, Humboldt-Universität Zu Berlin, and Berlin Institute of Health, Philippstrasse 12, 10115 Berlin, Germany; 7 Department of Chemical Biology and Bioimaging, Faculty of Chemistry, Wrocław University of Science and Technology, Wybrzeże Wyspiańskiego 27, 50-370 Wroclaw, Poland; 8 Department of Animal Developmental Biology, Institute of Experimental Biology, University of Wroclaw, Wroclaw, Poland; 9 Division of Histology and Embryology, Faculty of Medicine, Wroclaw Medical University, Wroclaw, Poland

**Keywords:** Betulin, Inflammation reduction, IL-6, COX-2, Amino acid Esters of Betulin

## Abstract

Betulin is a heavily studied natural compound for its use as an anticancer or pro-regenerative agent. The structural similarity between betulin to steroids gives rise to the idea that the substance may as well act as an anti-inflammatory drug. This study is the first to describe the anti-inflammatory properties of betulinic acid, betulin, and its derivatives with amino acids 1,4-diaminebutane (Dab), 1,3-diaminepropane (Dap), Ornithine (Orn), and lysine (Lys) on murine macrophages from lymphoma site. The compounds were compared to dexamethasone. To establish the response of the macrophages to the natural compounds, we tested the viability as well as sensitivity to the inflammatory signaling (IFNγR). IL-6 secretory properties and HSP-70 content in the cells were examined. Furthermore, we characterized the effects of compounds on the inhibition of cyclooxygenase-2 (COX-2) activity both in the enzymatic assays and molecular docking studies. Then, the changes in COX-2 expression after betulin treatment were assessed. Betulin and betulinic acid are the low-cytotoxicity compounds with the highest potential to decrease inflammation via reduced IL-6 secretion. To some extent, they induce the reorganization of IFNγR with nearly no effect on COX-2 activity. Conversely, Bet-Orn and Bet-Lys are highly cytotoxic and induce the aggregation of IFNγR. Besides, Bet-Lys reduces the activity of COX-2 to a higher degree than dexamethasone. Bet-Orn is the only one to increase the HSP-70 content in the macrophages. In case of IL-6 reduction, all compounds were more potent than dexamethasone.

**Supplementary Information:**

The online version contains supplementary material available at 10.1007/s10753-022-01756-4.

## INTRODUCTION

Betulin (BE) and betulinic acid (BA) are pentacyclic lupane-type terpenoids naturally occurring in the birch tree bark [1]. These compounds display anticancer, antiviral, antibacterial, antifungal, and anti-inflammatory properties, which have been described in the literature multiple times [2–4]. However, despite the high efficacy and safety of betulin, its use is limited due to low bioavailability and poor solubility in water [5, 6].

Inflammation plays a critical role in fighting against pathogens and neoplasms but also is involved in the pathological response towards unharmful agents. Various clinical disorders, such as psoriasis, chronic dermatitis, or atopic dermatitis, may be treated by reducing the inflammation in the skin tissue [7, 8]. Reduced chronic inflammation process in the tumor site decreases the accessibility of the tumor-propagation excretory factors, such as interleukins or prostaglandins [9–11]. Moreover, attenuation of the tumor site inflammation leads to the decrease in matrix-modulating proteins in the tumor microenvironment, thus preventing the tumor growth and infiltration of the neighboring tissues [12–16].

Best examples of cells that both release and self-depend on inflammatory factors are macrophages [17]. These may be involved in the formation of the lymphoma’s microenvironment. In this case, the attenuation of inflammation would benefit the lack of tumor progression and paracrine stimulation of the other cancer cells to replicate. In some cases, the high-dose dexamethasone is effective and leads to the arrest of cell replication and diminishes the size of the tumor. However, systemic therapy with steroids leads to a wide range of side effects—these include drug-induced Cushing syndrome, abnormalities in the lipid parameters of the blood, weight gain, and diabetes [18–20]. Therefore, the search for more safe and more effective steroid replacements is required.

Derivatives of BE and BA with higher bioavailability, which preserve their targeted activity or even outperform their precursors in that respect, are in high demand [21–23]. In the years 2009–2015, the affiliated research groups synthesized derivatives of BE—being amino acid esters of BE—characterized by significantly higher solubility in water compared to BE and BA. The previous reports present anticancer properties of these compounds [24–26] and their ability to stimulate collagen synthesis [27]. Currently, we are describing anti-inflammatory properties of amino acid esters of BE in relation to BE, BA, and a classic corticosteroid—dexamethasone. The structural resemblance of BE to dexamethasone indicates a similar mode of action of both compounds—as described and confirmed in 2021 [28]. Several other studies considered the application of the natural compounds to hematological malignancies, including lymphomas and leukemias [29–31]. The analyzed compounds came out as effective, which only encouraged the researchers to further evaluation.

The purpose of our studies was to assess the anti-inflammatory activity of amino acid esters of BE—BE-Lys-NH_2_ (ester with lysine), BE-Orn-NH_2_ (ester with ornithine), BE-Dap-NH_2_ (ester with 1,3-diaminepropane), and BE-Dab-NH_2_ (ester with 1,4-diaminebutane) in relation to BE, BA, and dexamethasone. In the beginning, we performed *in vitro* studies and assessed the immunomodulatory properties of BA, BE, and its derivatives and compared them with dexamethasone. We examined the secretion of IL-6 and IFNγR1 expression, thus the sensitivity towards the paracrine signal from the other immunocompetent cells. Furthermore, we analyzed the intracellular content of HSP-70—the intracellular protein involved in the negative regulation of NLRP3 inflammasome activation [32–34]. Moreover, we tested the activity of cyclooxygenase-2 (COX-2) following the incubation with the test drugs. Besides, we analyzed the cytotoxicity (MTT assay) induced by BE derivatives on P388D1 cells. In the end, we analyze the interactions between compounds and COX-2 with the cellular fingerprint approach, used to identify new biologically active compounds in combination with structural fingerprints [35]. Based on the experimental data, we propose the characteristics of each of the BE derivatives with respect to the immunological function of the macrophages.

## RESULTS

### Effects on P388D1 Cell Line

**Fig. 1:**
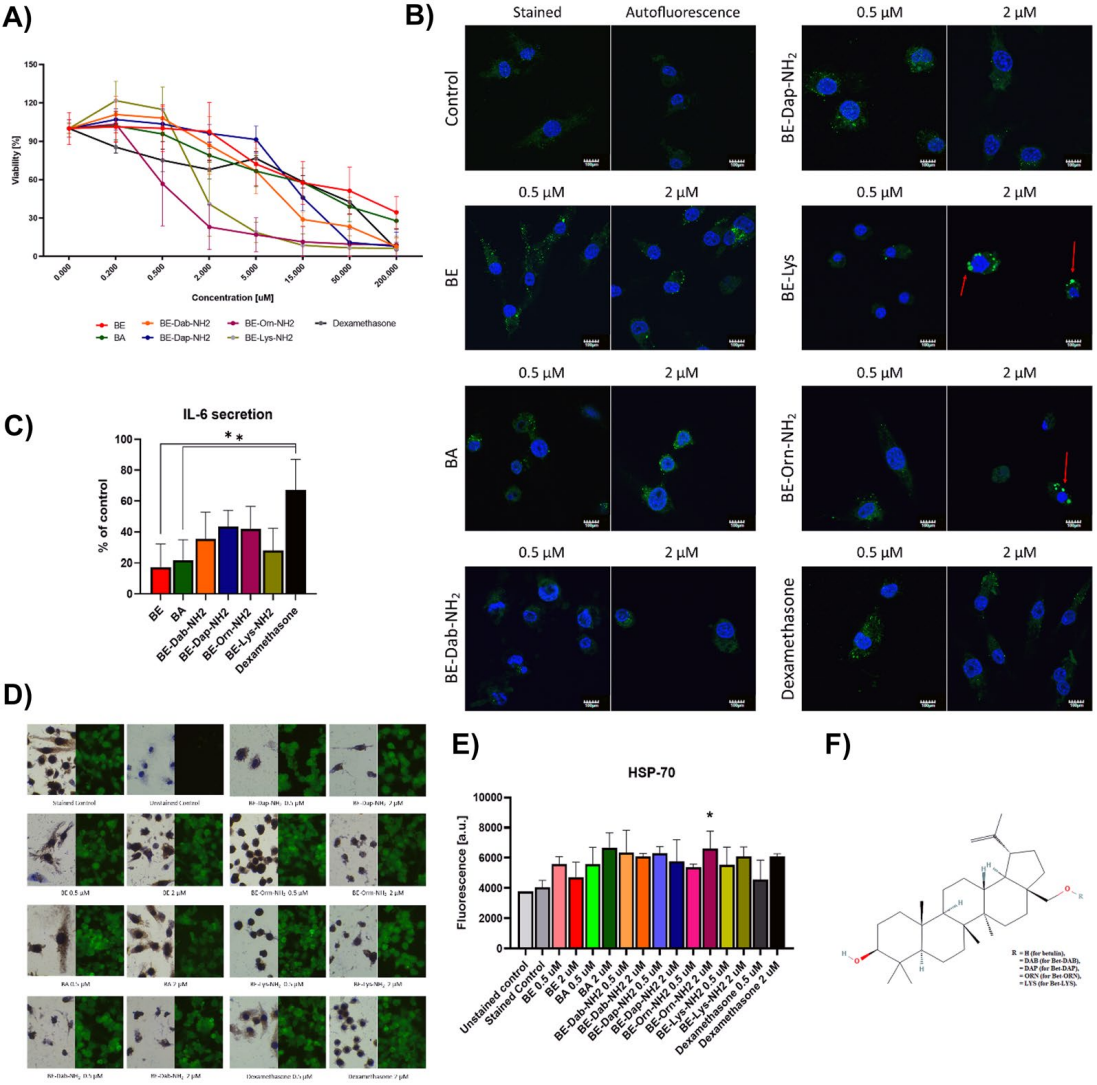
**A** MTT viability assay after 48-h incubation of P388D1 murine macrophages with BE and its derivatives. **B** IFNγR confocal microscopy localization studies; red arrows show the aggregates of the receptor; blue signal, DAPI (nuclei); green signal, AlexaFluor-488™ (IFNγR); scale bar 100 µm. **C** Interleukin 6 secretion ELISA assay analysis after treatment with 0.5 µM betulin and its derivatives. Dexamethasone was analyzed to show the standard inflammation attenuating agent. **D** Fluorescence studies of HSP-70 expression in P388 murine macrophages after 24-h incubation with betulin and its derivatives. ANOVA one-way analysis: *p < 0.05. **E** Quantitative fluorescence of COX-2 signal from **D**. **F** Structure of betulin and its derivatives; experiments performed in at least 3 biological replicates.

Figure 1A presents the mitochondrial activity of the murine macrophages (P388D1 cells) after 48-h incubation with BE, BA, BE-Dab, BE-Dap-NH_2_, BE-Orn-NH_2_, BE-Lys-NH_2_, and dexamethasone (compared to Fig. 1F). The compounds were tested in concentrations ranging from 0 to 200 µM. Both alkaline derivatives—BE-Orn-NH_2_ and BE-Lys-NH_2_—were characterized by the highest cytotoxic properties. Cytotoxicity of dexamethasone was comparable to the neutral betulin derivatives. Due to the relatively low cytotoxicity, for further experiments, 0.5 and 2 µM concentrations of betulin derivatives were chosen. The compounds are characterized by different cytotoxic properties; thus, some effects observed in subsequent experiments may be related to the toxicity of the specific substances (like BE-Orn-NH_2_).

On Fig. 1B, there might be observed small dot-like structures of the interferon-γ receptor in the cell. According to the literature, these structures may remain the IFNγR aggregates that were endocytosed from the cell membrane to the cytoplasm in the endocytosis vesicles. However, after treatment with Betulin and its derivatives, the increased signal objects enlarge and become highly visible. The effect is especially prominent in the macrophages treated with 2 µM BE-Orn-NH_2_ and BE-Lys-NH_2_. Curiously, BE-Dab-NH_2_ does not induce such effects in the P388D1 cells. The less cytotoxic derivatives do not show such enlarged IFNγR aggregates. Control samples show an even distribution of the IFNγ receptor. In case of dexamethasone, the effect is observed as well and may be compared to the effects of BE-Dap-NH_2_. Interestingly, there were observed changes specific for each of the compounds, between the 0.5 and 2 µM concentrations of the tested compounds [36].

Figure 1C presents the IL-6 secretory properties of the murine P388 macrophages treated with the non-toxic 0.5 µM solution of BA, BE, and its derivatives. For the experiment, we chose the concentration of the drug that did not induce any changes in cells’ viability to compare the amounts of IL-6 secreted by the same number of cells. Although each of the tested compounds decreased the excretion of IL-6 from the macrophages, it was only BE and BA, which induced statistically significantly (*p* < 0.05) lower secretion of IL-6 than 0.5 µM dexamethasone. The result proves that the potency of both BE and BA remains higher than dexamethasone in attenuating the immune response from the macrophages.

HSP-70 is an inflammation-related protein synthesized in the cytoplasm and secreted to the environment in response to the immune stimulus. Figure 1D presents the localization and fluorescence of HSP-70 signal in the lymphoma macrophages. Figure 1E shows its relative fluorescence signal. The only be which repeatedly increased the expression in the macrophages was 2 µM BE-Orn-NH_2_. There might be observed an interesting tendency—with the increase in compounds’ concentration, the HSP-70 signal increases in most cases. This might be explained in two ways—by the stimulation with compounds, the cell does not release HSP-70 to the extracellular compartment. The second option is that the cell increases the biosynthesis of the protein.

### COX-2 Inhibitory Assay

Cyclooxygenase-2 is a protein isoform present in macrophages. The enzyme is responsible for prostaglandins, leukotrienes, and lipoxin biosynthesis. The mentioned compounds are directly involved in the firing of the inflammation in the tissues affected by infection or the development of the tumor. Dexamethasone is a standard therapy agent to inhibit the inflammatory process by the inhibition of COX-2. To compare the standard agent with BA, BE, and its derivatives, we tested COX-2 activity after incubation with the drugs. All drugs were analyzed using the same two concentrations. Relatively high concentrations of the drugs were calibrated to the concentrations of dexamethasone, which induced the changes in COX-2 activity.

**Fig. 2:**
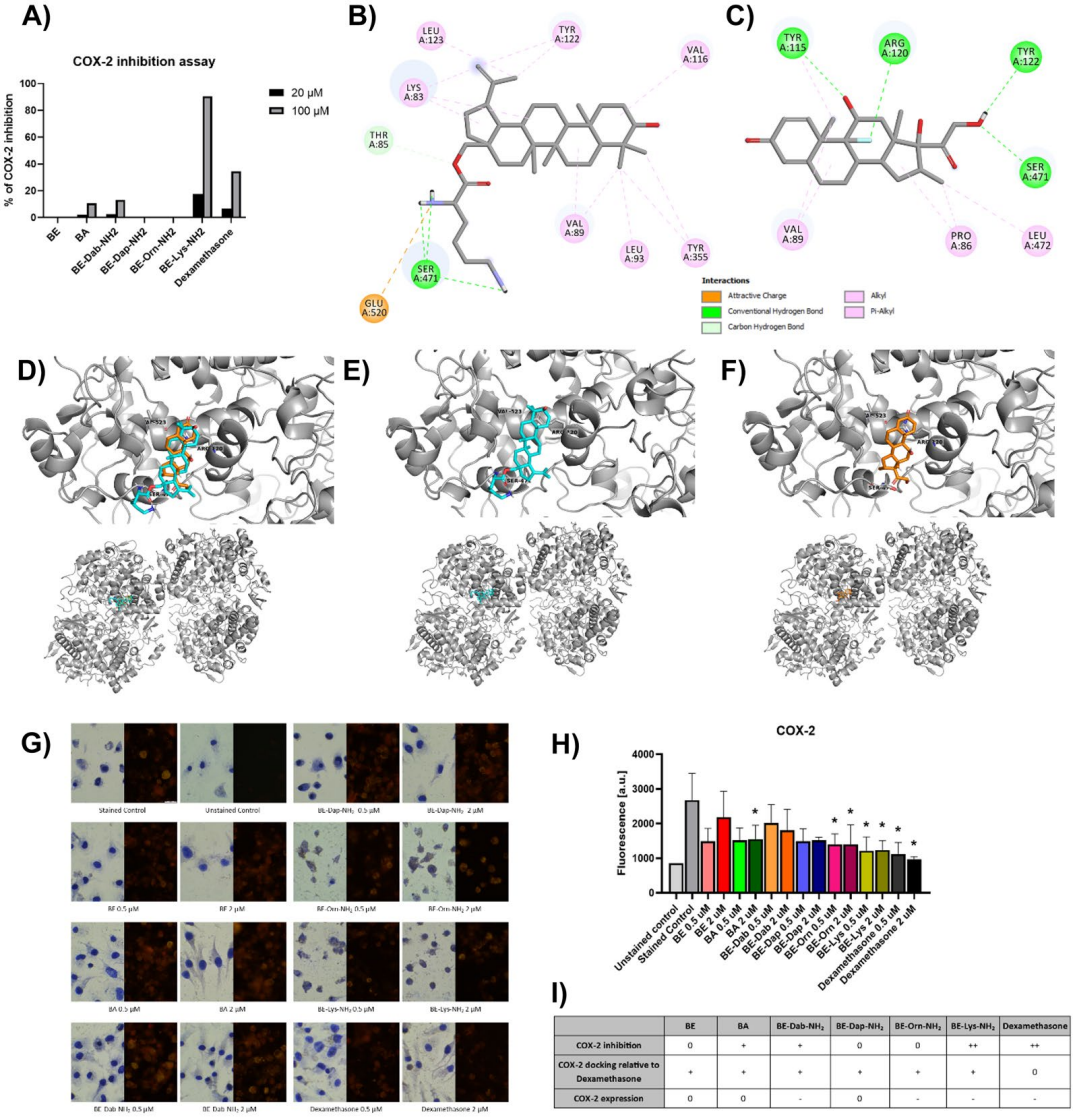
**A** COX-2 inhibition by BA, BE, and its derivatives in both 20 and 100 µM concentrations; **B** 2D representation of interactions between COX-2 (PDB: 4COX) and BE-Lys-NH_2_; **C** 2D representations of interactions between COX-2 (PDB: 4COX) and dexamethasone; **D** 3D representation of BE-Lys-NH_2_ (blue) and dexamethasone (orange) docked to COX-2 (gray, PDB: 4COX); **E** 3D representation of Bet-Lys (blue) docked to COX-2 (gray, PDB: 4COX); **F** 3D representation of dexamethasone (orange) docked to COX-2 (gray, PDB: 4COX); **G** immunocytochemistry and immunofluorescence studies of COX-2 expression on P388D1, the compounds were examined in both 0.5 and 2 µM concentrations; **H** quantitative fluorescence of COX-2 signal from G ANOVA; *p < 0.05.; **I** table summarizing the effects of each compound on the COX-2; experiments performed in at least 3 biological replicates.

Figure 2A shows the potency of BA, BE, and their derivatives in the inhibition of COX-2. There might be observed the highest drop-in activity after the application of BE-Lys-NH_2_. This compound interacts with Ser471 and forms the hydrogen bond, same as dexamethasone and with Tyr122, but in contrast to dexamethasone, it is an alkyl interaction, not a hydrogen bond (Fig. 2B and C). Both compounds also interact with VAL89. 3D visualization of BE-Lys-NH_2_ and dexamethasone (Fig. 2D, E, F) in complex with COX-2 shows that both compounds bind to the same place and thus may act in a similar manner. The other tested compounds were not as effective in the inhibition of the enzyme (Fig. 2A), even though BE-Dab-NH_2_, BE-Dap-NH_2_, and BE-Orn-NH_2_ interact with the same residues as BE-Lys-NH_2_ and dexamethasone, which are Ser471, Tyr122, and Val89 (Supplementary materials).

Analyses of interactions of co-crystallized ligand (indomethacin) with COX-2 show that this compound interacts with Ser530, Tyr355, Ser353, and Arg120 by forming hydrogen bonds. Those interactions were also mentioned as curtail by other authors in their docking studies [37–39]. But in case of BE-Lys-NH_2_, BE-Dab-NH_2_, BE-Dap-NH_2_, BE-Orn-NH_2_, and BE, only alkyl interaction with Tyr355 was present and in case of BA, hydrogen bond with Tyr355 (Supplementary materials).

To assess how the potential inhibition of COX-2 might affect its expression, we tested the enzyme’s expression with immunocytochemistry and immunofluorescence methods. In Fig. 2G and H, there might be observed the expression drop after treatment with the drugs that also inhibited the enzyme (BE-Lys-NH_2_ and dexamethasone). Table 2I presents all the effects on the COX-2.

## DISCUSSION

The presented studies had two main goals—to analyze the cytotoxicity towards P388D1 macrophages from the lymphoma site and to validate the potency of BA, BE, and its derivatives in the reduction of the inflammatory process. Moreover, we aimed to compare the response of the cells towards the natural compounds with the response towards dexamethasone, which is currently used for the mentioned purposes. In case of the cytotoxicity induced by compounds, we prove the high anticancer activity of BE-Orn-NH_2_ and BE-Lys-NH_2_. Both the analyzed hydrophilic derivatives were more cytotoxic than the corresponding compounds and dexamethasone. All the other analyzed compounds were cytotoxic to a similar extent. Due to the relatively low concentration of the drug in the tumor site and potentially fast metabolism, we chose the last non-toxic concentrations of the natural substances for further studies.

We analyzed the potency of 0.5 and 2 µM drugs on the localization and signal from HSP-70 and the localization and distribution of IFNγR. We proved that the cells incubated with BA, BE, and its derivatives do not change the cellular content of HSP70 nor its distribution (staining studies showed the mainly nuclear localization of the protein). Knowing that secretory HSP-70 may be involved in the propagation of inflammation, acting as the inflammation-related protein, we conclude that the lack of changes in HSP70 intracellular level relates to the lack of paracrine signal release and thus the lack of inflammation. Studies by Martine et al. prove that cellular HSP-70 attenuates inflammasome activity, and thus, its accumulation may act in an immunosuppressive manner [33]. The only compound to increase HSP-70 signal was 2 µM BE-Orn-NH_2_.

Our study also analyzed the expression and localization of IFNγR. In normal conditions, the receptor is present on the cell membrane and in the cytoplasm enclosed within the endocytic vesicles [36]. However, after treatment with BE and its derivatives, the receptor’s signal gathered in spot-like regions. According to the authors, the enhanced localization of IFNγR in the cytoplasm may relate to the receptors’ endocytosis and, therefore, the insensitivity of the macrophages towards the inflammatory signal from the other cells [36]. This time, BE-Lys-NH_2_ and BE-Orn-NH_2_ were the most effective. Both compounds in 2 µM concentrations induced the formation of the greatest signal from the receptor in the cytoplasm. Due to the molecular changes in IFNγR distribution after treatment with 0.5 µM substances, we chose to further analyze the compounds exclusively in this concentration of the drug and compare it with the control dexamethasone. Here, we analyzed the properties of 0.5 µM natural substances in the reduction of IL-6 secretion. According to the reduction in IL-6 secretion, each compound was more effective than dexamethasone. However, it was 0.5 µM BE and BA that induced the highest decrease. Various authors pointed out the relation between IFNγ and IL-6 signaling [40]. Regis et al. proved that IL-6 and IFNγ act differently on the T-cell neoplasms—namely, IL-6 induces apoptosis in STAT-3 depleted malignant cells [41]. Costa-Pereira et al. prove that an IFN-γ-like response to IL-6 may be observed when STAT3 is absent [42]. Kumari et al. show that IL-6 is deregulated in cancer and present in high concentrations in the tumor microenvironment, thus inducing all the hallmarks of cancer [43]. BA, BE, and its derivatives seem to cover all the potential complications of IL-6/IFNγ interplay in the tumor microenvironment.

BE and its derivatives proved their efficacy in the induction of cytotoxicity in cancer cells [3]. Moreover, the compounds were potent in regenerative medicine studies [27]. However, no other study considered the application of the analyzed natural compound as an anti-inflammatory agent. In general, at the same concentration, all the analyzed compounds were more effective in the inhibition of macrophages’ function. However, the detailed characteristics of each derivative were heavily different. Basic derivatives (LYS and Orn) were highly cytotoxic. The compounds both induced the aggregation (and probably the insensitivity of the cells towards IFNγ signal) of the IFNγR and reduced the expression of COX-2 to the highest extent. Also, BE-Lys-NH_2_ was the one to inhibit the activity of the enzyme in a similar mechanism to the dexamethasone. In the case of BE-Orn-NH_2_, it was the only one to increase the cellular expression of HSP-70 significantly. In this case, the cells might avoid releasing the inflammation-related protein. Combining with the potency of the basic drugs to reduce the secretion of IL-6, we may assume that both of them would reduce inflammation. On the other hand, there are natural compounds. Both of the compounds were highly potent in reducing the IL-6 secretion in comparison to dexamethasone. Combining that with the relatively low cytotoxicity, the drugs may be potentially used as an alternative for dexamethasone in the reduction of inflammatory process. Nearly lack of the effect on COX-2 nor the HSP-70 expression could potentially reduce the side effects normally caused by dexamethasone. The least interesting from the point of the study was BE-Dap-NH_2_ and BE-Dab-NH_2_, which only advantage over dexamethasone seems to be the hydrophilic nature in case of potential oral administration. Properties of BA, BE, and their derivatives are summarized in Table 1.

**Table 1:** Properties of BE, BA, BE-Dab-NH_2_, BE-Dap-NH_2_, BE-Orn-NH_2_, BE-Lys-NH_2_, and Dexamethasone as the Reference Drug. In the table, “ + ” indicates the increase, “ + / − ” indicates the slight increase, “ − ” indicates the decrease, and “0” indicates the lack of effect

	**HSP-70 signal**	**IFNγR bodies**	**Cytotoxicity**	**IL-6 secretion attenuation**	**COX-2 inhibition**
**Betulin**	0	0	0	+	0
**Betulinic acid**	0	0	0	+	+ / −
**BE-Dab-NH**_**2**_	0	0	0	+ / −	+ / −
**BE-Dap-NH**_**2**_	0	0	0	+ / −	0
**BE-Orn-NH**_**2**_	+	+	+	+ / −	0
**BE-Lys-NH**_**2**_	0	+	0	+ / −	+
**Dexamethasone**	0	0	0	+	+

Our study shows the potential of betulin derivatives in reduction of inflammation and induction of cell death in murine macrophages. The limitation of the study is that all the compounds were tested only in a single cell line. However, other studies prove similar tendencies, showing the high potential of betulins as the anti-inflammatory agents [44].

## MATERIALS AND METHODS

### Cell Culture

The murine macrophages cell line P388/D1 (ATCC^®^ CCL-46™) was obtained from the lymphoma site of the mouse. Cells were cultured as a monolayer in Dulbecco’s modified Eagle’s medium (DMEM, Sigma-Aldrich, St. Louis, MO, USA) mixed with RPMI-1640 medium (Sigma-Aldrich, St. Louis, MO, USA) in 1:1 ratio. The medium was supplemented with 10% fetal bovine serum (FBS, Sigma-Aldrich) and an antibiotic (streptomycin/penicillin). The cells were incubated at 37 °C in a humidified atmosphere containing 5% CO_2_. The cells were characterized by adherent lymphoblastic morphology. When needed, the cells were washed with PBS and removed by trypsinization (0.025% trypsin and 0.02% EDTA; Sigma-Aldrich).

### Drug Preparation

Betulin (BE) and betulinic acid (BA) were purchased from Sigma company (Sigma, Poznan, Poland). Betulin derivatives were synthesized by prof. Marcin Drąg and Marcin Poręba on Wrocław University of Technology according to the already published paper [25]. Dexamethasone was used in the form of the injection drug (Dexaven^®^, 4 mg/ml, Bausch Health, Ireland). Due to the fact that Dexaven^®^ is composed of dexamethasone sodium phosphate, the *in vitro* experiments were carried out after 12-h incubation in plasma-containing culture medium, which is greater than the time required for drug hydrolysis in plasma (according to a paper by Samtani et al.) [45].

### MTT Viability Assay

Cells’ viability after 48-h incubation with test compounds was analyzed with mitochondrial activity assay (MTT). The culture medium was removed from each well, and 100 µL of 0.5 mg/mL MTT (3-(4,5-dimethylthiazol-2-yl)-2,5-diphenyltetrazoliµM bromide, Sigma-Aldrich) solution in PBS buffer was added. After 2 h of incubation at 37 °C, acidified isopropanol (100 µL, 0.04 M HCl in 99.9% isopropanol) was added to dissolve the formazan crystals. The samples were fully dissolved by the pipet mixing technique. The absorbance of each well was measured at 570 nm using a multiplate reader (GloMax, Promega, Walldorf, Germany). The results were expressed as the percentage of viable cells relative to untreated control cells.

### Fluorescent Studies

Anti-HSP-70 antibody (sc-32239, Santa Cruz, USA) was applied to assess the level of HSP-70 in P388D1 cells after incubation with BA, BE, and its derivatives. Anti-COX-2 antibody (C6827, Thermo Fisher Scientific, Waltham, MA, USA) was applied to assess the level of COX-2 in P388D1 cells after incubation with BA, BE, and its derivatives. The cells were incubated on in Petri dishes for 24 h to attach. Afterward, the culture medium was replaced with a medium containing the analyzed compounds. Twenty-four-hour incubation was performed. Afterward, the cells were washed with PBS and fixed with 4% formalin. As soon as three replicates were performed, the single staining procedure was performed to obtain similar fluorescence between the same samples from different replicates. The samples were initially treated with Triton-X100 for 5 min to permeabilize the membranes. Following, the incubation with FBS was performed for 1 h. Next, the cells were washed with Triton-X100, and a primary antibody was added for 1-h incubation. Following, the cells were washed with PBS and the secondary antibody was added for 1-h incubation. In the end, the samples were washed with PBS. The samples were observed on the Olympus IX53 microscope (40 × , Olympus, Tokyo, Japan) after blue and green laser excitations (depending on the fluorophore). The fluorescence of cells on each sample was analyzed in the CellProfiler software. Each sample consisted at least three photographs. All the data was plotted and statistically analyzed.

### Immunocytochemical Staining

Immunocytochemical staining aimed to assess the expression and distribution of HSP-70 in P388PD1 cells after the incubation with BA, BE, and its derivatives. The cells were incubated on the 10-well microscopy slides overnight. Afterward, the media was replaced with culture media containing tested compounds in 0.5 and 2 µM concentrations. After 24 h, the cells were washed with PBS. Then, cells were fixed in 4% formalin. Afterward the cells were washed in PBS once again. Hydrogen peroxide block was added for 10 min. After the time, the cells were washed in PBS with 1% Triton X. Protein block agent was incubated with the cells for 10 min. Furthermore, the cells were incubated with the first-order antibodies for 24 h in 4 °C. Afterward, the cells were washed with PBS and 1% Triton X. Mouse Complement agent was added for 10 min. Afterward, the cells were washed with PBS with 1% Triton X, and Dab mixture (1 Dab:50 Dab substrate) was applied for 10 min in the dark. Next, the cells were washed for 10 min in distilled water. After that, the samples were stained with hematoxylin for 1 min. The excess of the stain was washed with water for 30 min. The cells were dehydrated by incubation for 5 min in each of ethanol solutions: 50%, 60%, 70%, 80%, 90%, and 96%. Xylene I and II were used in the last step of the preparation of the samples. In the end, DPX was used to mount the glass top of the sample. The samples were observed on the light microscope (Olympus BCX43, Tokyo, Japan). Plan-Apochromat 40 × and 100 × objectives (Olympus) were used to capture the images.

### Confocal Microscopy Studies of IFNGR1

Phospho-IFNGR1 staining (1:500, PA5-38,504, Thermo Fisher) was performed to visualize the distribution of the receptor in the murine macrophages following the incubation with BA, BE, and its derivatives. The cells were incubated on cover glasses in Petri dishes overnight to attach. After that, 48-h incubation with test compounds was performed, and then, cells were fixed in 4% formalin solution. The samples were treated with Triton-X100 for 5 min to permeabilize the membranes. Following, the incubation with FBS was performed for 1 h. Next, the cells were washed with Triton-X100 and a primary antibody was added for 1-h incubation. Following, the cells were washed with PBS, and the secondary antibody (Alexa Fluor 488) was added for 1-h incubation. In the end, the samples were washed with PBS. Fluoroshield™ with DAPI (4,6-diamidino-2-phenylindole) was applied to visualize the nuclei and to mount the cells. The samples were observed on the Olympus FluoView FV1000 confocal laser scanning microscope (Olympus, Tokyo, Japan).

### ELISA Assay for IL-6 Release Quantification

Secretory properties of the P388D1 cells after 48-h incubation 0.5 µM with BA, BE, and its derivatives were analyzed with Mouse IL-6 ELISA Kit (ab100712, Abcam). Cells were seeded in 20,000 cells/well density on the 96-well plate. After 24-h adhesion-required time, the cells were treated with BA, BE, and its derivatives for 48 h. Afterward, the culture medium was collected from each well and stored at − 20 °C, until three replicates of the experiment were performed. When the whole material was gathered, the medium was transferred to a 96-well plate coated with antibodies specific for Mouse IL-6. The wells were washed afterward, and a biotinylated anti-Mouse IL-6 antibody was added. Next, after washing away, the unbound antibody, the HRP-conjugated streptavidin, was pipetted to the wells. In the end, the stop solution was added to each well, so the color changed from blue to yellow—proportionally to the total amount of IL-6 in each well. The absorbance was measured at 450 nm, and the data was plotted.

### COX-2 (Ovine) Colorimetric Inhibitor Screening Assay

COX-2 peroxidase activity was measured by the colorimetric method where the oxidized form of N,N,N′,N′-tetramethyl-p-phenylenediamine (TMPD), which is the substrate for most enzymes with peroxidase activity, was changed. COX peroxidase component converts arachidonic acid to the reduction form PGG2 (prostaglandin G2) and then to corresponding alcohol PGH2 causing the oxidation of TMPD and resulting in a change in color measured at 590 nm. Ten microliters of the test substance at a concentration of 20 µM and 100 µM was added to the incubation mixture consisting of 150 µL of Tris–HCl buffer (0.1 M, pH 8), 10 µL of heme, and 10 µL of the COX-2 enzyme. The mixture was incubated for 5 min at 25 °C, 20 µL of colorimetric substrate solution (TMPD) was added, followed by 20 µL of 1.1 mM arachidonic acid, the reaction was incubated for 2 min at 25 °C, and the absorbance was measured at 590 nm using BioTek spectrophotometer microplate reader MQX200 (BioTek, Winooski, VT, USA).

All of the test samples were dissolved in DMSO. DMSO alone was used as a blank. The inhibitory activity was expressed as percentage inhibition calculated as (absorbance of the control minus absorbance of the test drug)/(absorbance of the control) × 100%. Experiments were done in triplicate.

### Molecular Docking Studies

Molecular docking was performed with the CB-dock online server [46]. This is a blind docking server, which predicts binding sites of proteins and also calculates docking scores using AutoDock Vina docking software [47]. Protein structures were downloaded from RCSB PDB database [48]. Structures of compounds were either downloaded from the PubChem database or were created with Avogadro Software [49]. Cyclooxygenase-2 (COX-2) with PDB code 4COX was chosen for docking. Crystal structure 4COX was available in complex with co-crystallized ligand—indomethacin. First, the co-crystallized ligand was re-docked, and the enzyme’s binding site was identified. Additionally, dexamethasone was docked for a comparison of the binding mode. The results of the molecular docking were analyzed, and 2D interaction diagrams of the best-docked pose were created with BIOVIA Discovery Studio Visualizer [50]. For the generation of 3D visualization of protein–ligand complexes of chosen compounds, PyMOL software was used [51]. The supplementary materials section included the results of all docking studies.

### Statistical Analysis

Viability experiments were performed in at least 3 biological replicates. IFNGR1 was examined independently in immunofluorescence (confocal and fluorescent microscopy) and immunocytochemical studies. ELISA for IL-6 assessment was performed in 3 biological replicates. HSP70 staining studies were performed in at least 3 replicates, involving immunofluorescence and immunocytochemical staining. Data presented in the paper shows the mean of the obtained photographs. In the paper, the authors presented the staining, which was the most consistent with other data.

Data were expressed as mean ± SD and analyzed by two-way ANOVA (in GraphPad Prism 8), with *p* < 0.05 being considered statistically significant.

## CONCLUSIONS

BE and its derivatives may be considered potential anti-inflammatory agents. All of the compounds are more effective in the decrease of IL-6 secretion. However, the efficacy of COX-2 inhibition mainly was connected with Bet-Lys. Besides, after stimulation with basic betulin derivatives (Bet-Lys, Bet-Orn), the cells were less sensitive to the IFNγ stimulation, due to the polymerization of the interferon receptor. Moreover, the hydrophilic drugs were cytotoxic towards cells in much lower concentrations than the hydrophobic BE. In summary, BE and its derivatives present an interesting alternative to dexamethasone in the attenuation of macrophages’ inflammatory response. However, further *in vivo* studies are required to validate the potential clinical use of the natural compounds.

## Supplementary Information

Below is the link to the electronic supplementary material.

Supplementary file1 (DOCX 637 KB)

## Data Availability

The datasets generated during and analyzed during the current study are available on request to the authors.
