# Supplementary material for: Betulin and Its Derivatives Reduce Inflammation and COX-2 Activity in Macrophages
Source: Inflammation. 2022 Oct 25;46(2):573–83. doi: 10.1007/s10753-022-01756-4 (PMC10024662; doi:10.1007/s10753-022-01756-4)
Supplement: Supplementary file 1 — Supplementary file1 (DOCX 637 KB) [file 10753_2022_1756_MOESM1_ESM.docx]

# Docking to COX-2 (PDB: 4COX)

| Compound | 2D interactions diagram | Vina score [kcal/mol] |
| --- | --- | --- |
| Dexamethasone | 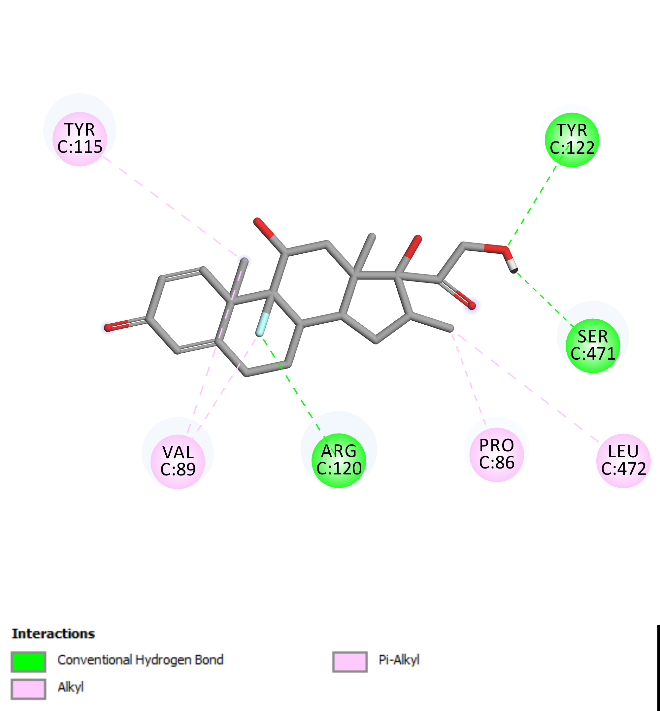 | -8.7 |
| Betulin | 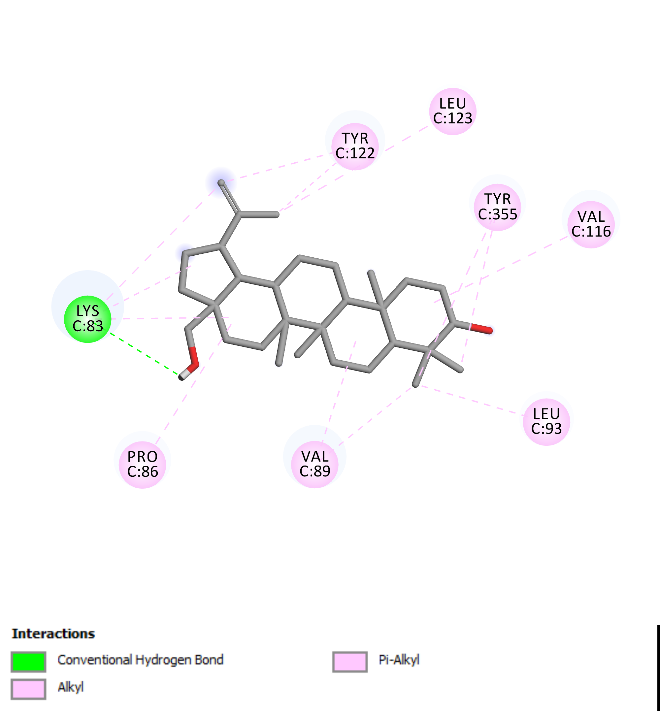 | -8.0 |
| Betulinic acid | 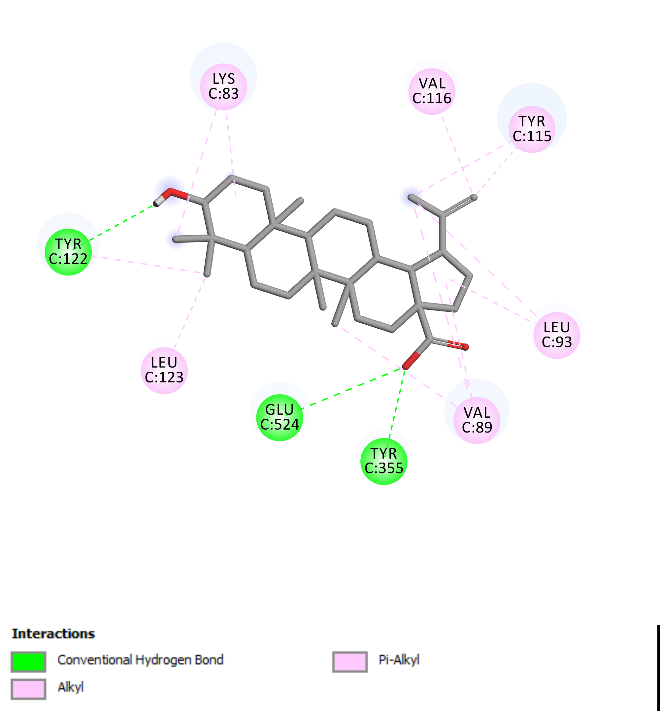 | -8.4 |
| BE-Dab-NH_2_ | 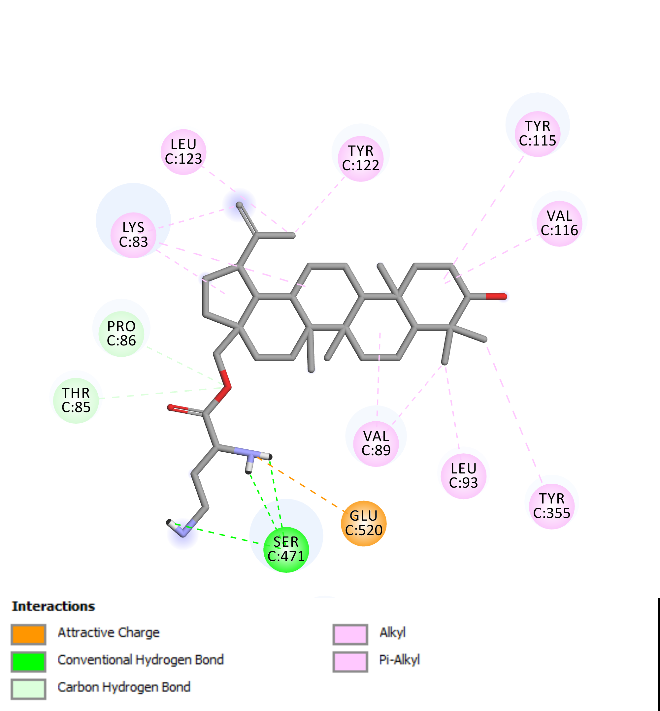 | -7.6 |
| BE-Dap-NH_2_ | 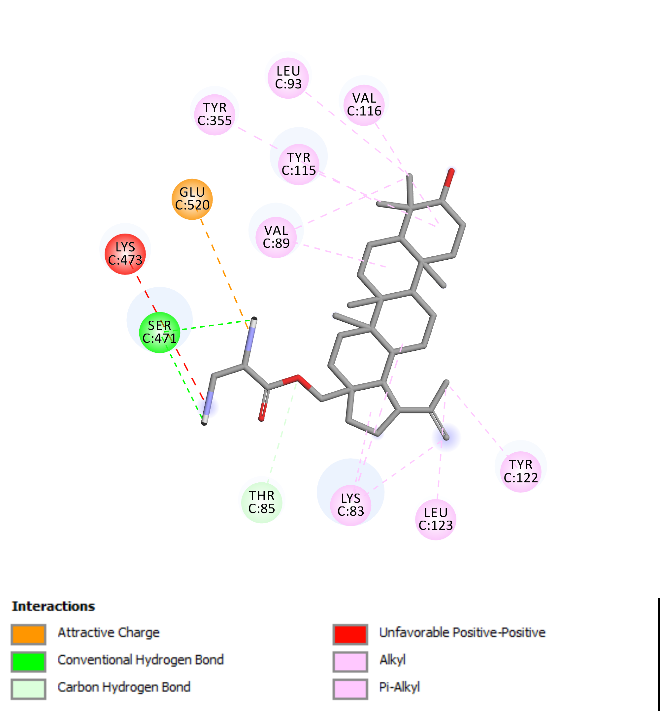 | -7.7 |
| BE-Orn-NH_2_ | 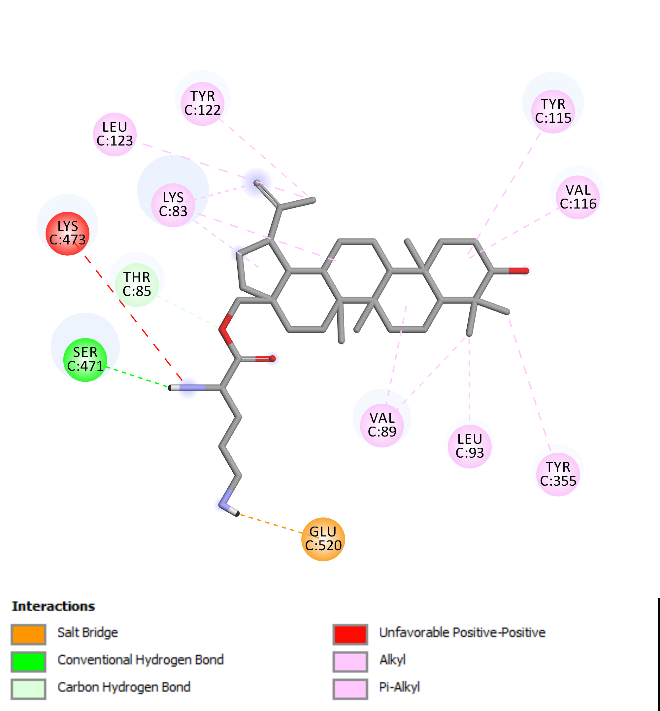 | -7.7 |
| BE-Lys-NH_2_ | 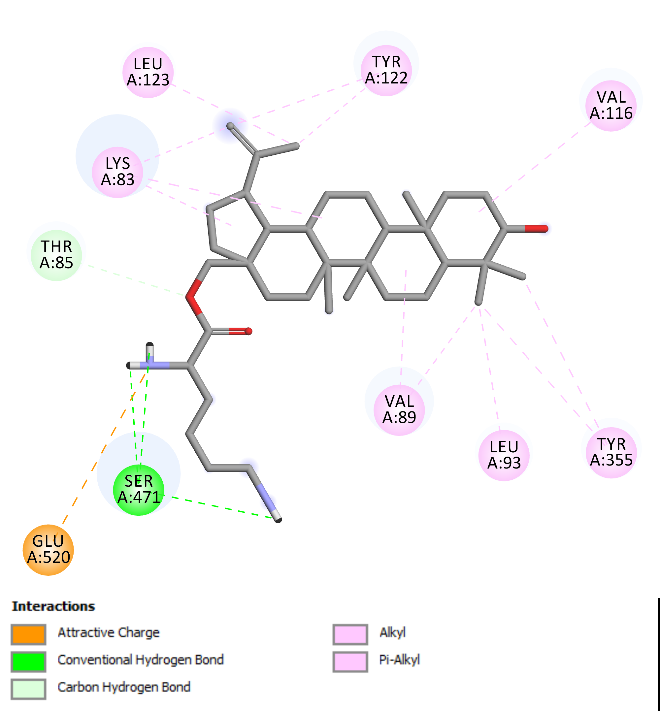 | -7.6 |
| Indomethacin | 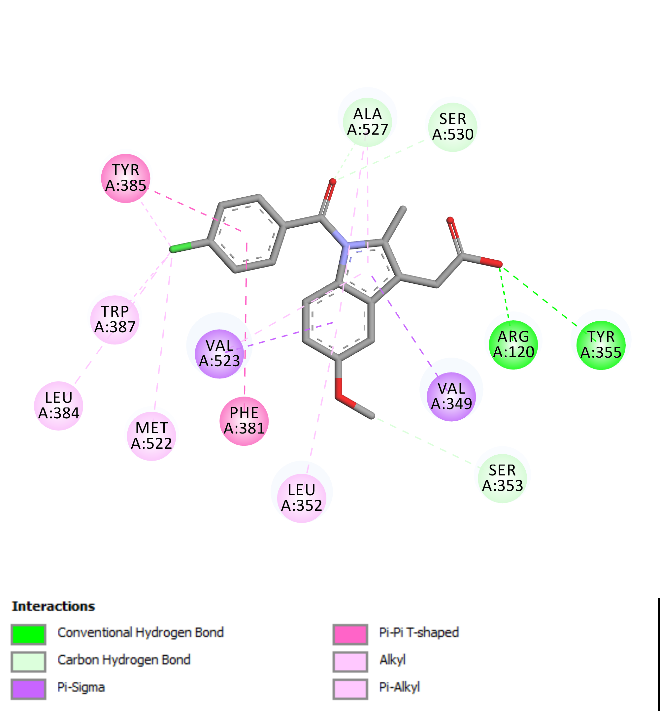 | -8.7 |
